# The Role of Somatic Maturation on Bioimpedance Patterns and Body Composition in Male Elite Youth Soccer Players

**DOI:** 10.3390/ijerph16234711

**Published:** 2019-11-26

**Authors:** Francesco Campa, Analiza Monica Silva, Vincenzo Iannuzzi, Gabriele Mascherini, Luca Benedetti, Stefania Toselli

**Affiliations:** 1 Department of Biomedical and Neuromotor Sciences, University of Bologna, 40126 Bologna, Italy; stefania.toselli@unibo.it; 2 Exercise and Health Laboratory, CIPER, Faculdade de Motricidade Humana, Universidade de Lisboa, 1499-002 Cruz-Quebrada, Portugal; analiza.monica@gmail.com; 3 Department of Statistical Sciences, University of Bologna, 40126 Bologna, Italy; vincenzo.iannuzzi@studio.unibo.it; 4 Department of Experimental and Clinical Medicine-Sports Medicine and Exercise Unit, University of Florence, AOUC, Careggi, 50139 Florence, Italy; gabriele.mascherini@unifi.it; 5 School of Pharmacy, Biotechnology and Sport Science, University of Bologna, 40127 Bologna, Italy; lucaben759@gmail.com

**Keywords:** BIVA, R–Xc graph, peak at velocity, phase angle, vector length

## Abstract

The purpose of this study was to examine the influence of chronological age (CA) and somatic maturation on body composition (BC) and bioimpedance parameters in male elite soccer players. BC and bioimpedance variables were measured in a sample of 249 players aged 9−18 years of age and registered in two professional Italian soccer teams. Results from segmental analysis showed transition time points where the influence of CA and somatic maturation on bioimpedance patterns and BC characteristics increased or subsided. The accelerated phases were assessed for fat free mass, total body water, and upper muscle area, with a starting time point at approximately −2.00 years from peak at velocity (YPHV), and for body cell mass, whose developmental tempo sped up around −1.00 YPHV. An increase in the rate of development was also observed close to −2.00 YPHV for phase angle (PA), although without accelerated phases. From a CA point of view, significant slope changes were found for all BC and bioimpendance variables, except for the calf muscle area. Although the starting points and the span of the accelerated phases were different, they subsided or disappeared at ~ 15 years, except for PA, whose growth waned at ~ 17 years.

## 1. Introduction

The evolution of a successful athlete is multifaceted in nature. Various factors including technical skills, physical performance, environmental circumstances, and social conditioning contribute to the development of this process. Over time, complex interactions and a nonlinear progression among these factors contribute to elite level achievement in youth athletes [1,2].

Youth soccer players that show advanced somatic maturation are often preferred over their late-maturing peers. This occurs due to the fact that during talent identification, they present better physical characteristics and performance, especially those around the age of peak velocity [3,4,5,6].

There is a close relationship between performance parameters and body composition (BC). BC-related parameters can be used in talent identification, they vary within sports and help to build player position profiles [7,8,9]. In particular, BC features derived by anthropometric measurements, such as fat mass (FM), upper fat area (UFA), thigh fat area (TFA), calf muscle area (CMA) are strongly associated with repeated sprint performance ability in elite soccer players [8]. Additionally, a high fat mass percentage (%FM) negatively affects endurance, strength and power qualities, which are assessed by soccer-specific tests [8,10]. Indeed, recent studies have shown that advanced biological maturity, within single year chronological age (CA) categories, is associated with advantages in body size and fat free mass (FFM) in adolescent athletes [5,11,12].

It is well known that using prediction equations can present some limitations when evaluating BC, especially in the assessment of younger subjects [13]. In this regard, an interesting approach commonly used in the sports field is bioelectrical impedance vector analysis (BIVA) [14,15,16,17,18,19]. This method plots the impedance parameters (resistance (R) and reactance (Xc)) standardized for the subject’s height on the R–Xc graph as a single vector [20]. Recently, different studies compared repeated BIVA, dual X-ray absorptiometry and body fluids measurements and showed the ability of BIVA to evaluate changes in BC by studying the vector displacements and BC variables, e.g., total body water (TBW), %FM and body cell mass (BCM) in athletes over a season in athletes [21,22]. Bioelectrical phase angle (PA), calculated as the arc tangent of Xc/R × 180°/π, is the most significant predictor of the extracellular to intracellular water ratio, and it has been shown to be positively correlated with chronological age because of the increase in BCM and body size that occur during biological maturation [23]. Furthermore, Koury et al. [24] demonstrated that early maturing soccer players have higher PA than those categorized as maturing late or on time considering the stage of skeletal maturity, thus highlighting the influence of delayed skeletal maturity on BIVA patterns.

To the best of our knowledge, there is limited data available on bioelectric and BC changes in relation to age and maturity status in youth elite level soccer players. As suggested by Towlson et al. [25], previous studies have attempted to explain the development of various performance features related to maturation using classic polynomial regression models to determine nonlinear relationships with regression parameters that are not directly interpretable. In fact, these experimental approaches do not specifically consider transition points in the relationship between somatic maturation and physical parameters.

Therefore, this study aimed to examine the influence of CA and somatic maturation on bioelectrical and BC parameters, by identifying the time points at which the influence of maturation increases, subsides, or ceases to exist on examined variables in soccer players. Another goal was to observe the age-related differences in BIVA vector displacements over the R–Xc graph in a large sample of male elite youth soccer players aged from 9 to 18 years of age.

## 2. Materials and Methods

### 2.1. Participants

A cross-sectional study was conducted on 249 elite male youth soccer players. The participants were recruited from two different youth academies, each of which is associated with an Italian professional soccer team. Non-injured players at the time of testing from the U10 to U19 age categories of the elite level teams were eligible for participation in this study. The testing procedures were performed during the first part of the preparation period of the 2019–2020 season (August 2019). Participation in the study was voluntary and all of the participants and their parents or legal guardians were properly informed regarding the risks and benefits of the study prior to any data collection and signed an institutionally approved informed consent document. The project was conducted in accordance with the guidelines of the Declaration of Helsinki and was approved by the local Bioethics Committee of the University of Bologna (Ethical Approval Code: 25027; dated 13 March 2017).

### 2.2. Body Composition

Body mass index was calculated as the ratio of body weight to height squared (kg/m^2^). Skinfold thickness at eight sites (biceps, triceps, subscapular, supraspinal, suprailiac, lateral calf, medial calf, and thigh) were measured to the nearest 0.1 mm using a Lange skinfold caliper (Beta technology Inc., Cambridge, Maryland). The muscle area of the thigh (TMA), calf (CMA) and upper arm (UMA), and the fat area of the thigh (TFA), calf (CFA) and upper arm (UFA) were calculated according to Frisancho [26]. The %FM, FFM, TBW, and BCM were predicted using the software Bodygram^®^ (Akern Srl., Pontassieve, Florence, Italy).

### 2.3. Bioelectric Impedance Vector Analysis

The impedance measurements were performed with bioimpedance analysis (BIA 101 Anniversary, Akern, Florence, Italy) using an electric current at a frequency of 50 kHz. Measurements were made using four electrical conductors; the subjects were in the supine position with a leg opening of 45° compared to the median line of the body and the upper limbs, distant 30° from the trunk. After cleansing the skin with alcohol, two Ag/AgCl low impedance electrodes (Biatrodes Akern Srl, Florence, Italy) were placed on the back of the right hand and two electrodes were placed on the corresponding foot [27]. To avoid disturbances in fluid distribution, athletes were instructed to abstain from foods and liquids for ≥4 h before the test. Athletes consumed a normal breakfast at 07:00 and the measurements were taken at 11:00. Vector length (VL) was calculated as (adjusted R^2^+adjusted Xc^2^)^0.5^ and PA as the arctangent of Xc/R x 180°/π. BIVA was carried out using the classic methods, e.g., normalizing R (Ω) and Xc (Ω) for height in meters [20]. Elite male soccer players bioelectrical specific values [28] were used as a reference to build the 50%, 75%, and 95% tolerance ellipses on the R–Xc graph.

### 2.4. Somatic Maturation

All anthropometric data was collected by a specifically trained physician. Height, sitting height, body weight were collected with participants wearing only shorts. Height and sitting height were recorded to the nearest 0.1 cm with a standing stadiometer (Raven Equipment Ltd., Great Donmow, UK) and body weight was measured to the nearest 0.1 kg with a high-precision mechanical scale (Seca, Basel, Switzerland). Leg length was derived as the difference between height and sitting height. An estimation of the years from peak height velocity (YPHV), which is an indicator for the adolescent growth spurt, was made using Mirwald’s equation for boys, which is able to predict maturity offset in youth athletes [29,30].

Maturity offset = –9.236 + 0.0002708 (leg length × sitting height) – 0.001663 (age × leg length) + 0.007216 (age × sitting height) + 0.02292 (weight: height)

Since maturity offset represents the time before or after peak height velocity (PHV), YPHV were calculated by subtracting age at PHV from CA.

### 2.5. Statistical Analysis

The mean ± standard deviation was calculated for each variable. The players whose YPHV values were out of the tolerance limits of the somatic maturation prediction equations (±4 years) [25,29] were excluded (n = 7). Then, simple linear regressions of the dependent variables (BC and bioelectric impedance data) vs. explanatory variables (CA and YPHV data) were empirically investigated and tested for changes in the response variables’ slope (Davies test) and for the existence of breakpoints (Pscore test). In order to identify breakpoint(s) where a change in the slope of the targeted variables is observed, we performed a segmented regression analysis using the “segmented” package (v 1.0.0) [31,32], and for each individual regression we selected the model with the lower Bayesian information criterion value. Delta method and sandwich estimator for the standard errors were used to compute 95% confidence interval (CI) of the breakpoint estimates. Slope coefficients estimates and the related 95% CIs were reported, and significant slopes were detected using alpha set at 0.05. Finally, the r^2^ statistic was computed to quantify the amount of variability explained by each segmented regression model. Statistical analysis was performed using the software R (v 3.5.2). (R Foundation, Vienna, Austria).

## 3. Results

Descriptive statistics of the players grouped by age categories are shown in Table 1.

**Figure 1:**
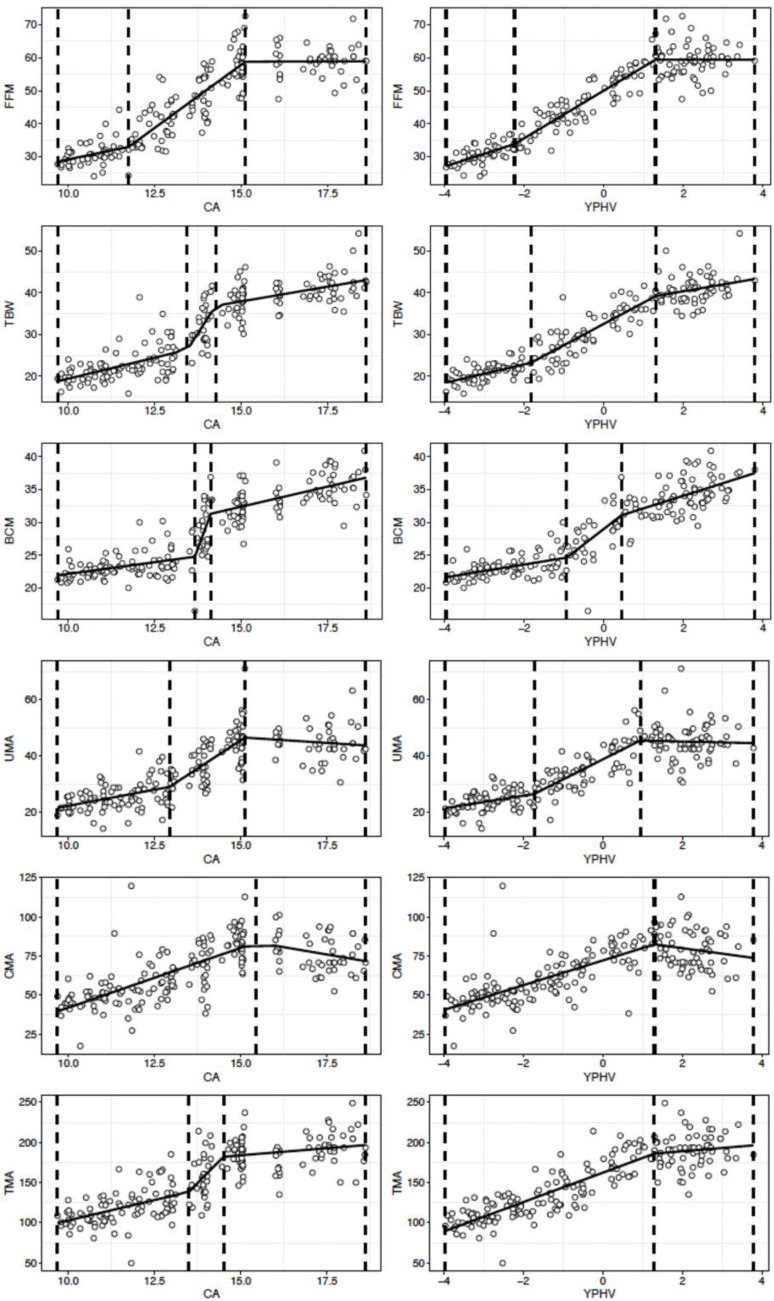
Body composition variables development according to player chronological age (years) and somatic maturity (YPHV). YPHV = years from peak high velocity.

**Figure 2:**
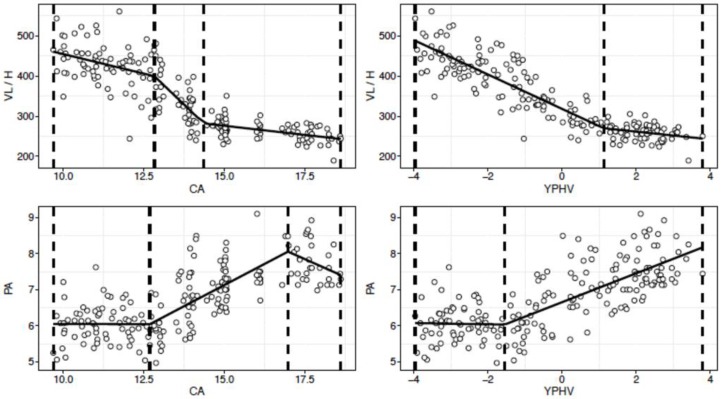
Bioimpedance values development according to player chronological age (years) and somatic maturity (YPHV).

We identified non-constant linear regression parameters in the linear predictor (Davies test: *p* < 0.05) and the existence of at least one breakpoint (Pscore test: *p*-value *p* < 0.05) for all bioelectrical and BC variables, except for %FM, UFA, CFA, and TFA (Table 2 and Table 3, Figure 1 and Figure 2).

### 3.1. Body Composition Parameters

For FFM, similar trajectories were observed for the CA and YPHV models. In each of the two individual regressions, two breakpoints were identified: 11.7 and 15.1 years when FFM was modeled against CA, and −2.2 and 1.3 when modeled against YPHV (Table 3). In particular, the FFM increased faster when CA and YPHV values were between the two estimated breakpoints, with annual growth rates of 7.6 kg/y and 7.2 kg/y, respectively. Furthermore, for both models the slope parameter was not significant (*p* = 0.48–0.50) after the second breakpoint and a plateau was observed.

Also, two breakpoints were identified for TBW in each of the two segmented regressions. Increases in TBW were greater between 13.4 and 14.2 years or −1.8 and 1.3 YPHV, with annual growth rates of 12.5 kg/y and 5.1 l/y, respectively. For both FFM and TBW, model strength was higher in YPHV (r^2^ = 0.91) than in CA (r^2^ = 0.84).

For BCM, the breakpoint estimates are as accurate as the other variables, but the 95% CIs of the two adjacent breakpoints overlapped. Significant growth rates were observed in both BCM-CA and BCM-YPHV models. However, the developmental gains were greater between 13.6 and 14.1 years, or between −0.9 and 0.4 YPHV, with annual growth rates of 14.1 kg/y and of 4.6, kg/y, respectively. The model strength was slightly higher in YPHV (r^2^ = 0.85) than in CA (r^2^ = 0.83).

For UMA, the segmented regression identified two trajectory changes in the rate of player development for both the CA (r^2^ = 0.72) and YPHV (r^2^ = 0.76) model. The greatest gain was observed between 12.9 and 15.1 years, or between −1.7 and 0.9 YPHV, with annual growth rates of 7.9 cm^2^/y and 7.1 cm^2^/y, respectively. After the second breakpoint the slope was not significant (*p* = 0.22–0.37) and the trajectory plateaued.

For CMA, annual growth rates were estimated at 7.6 cm^2^/y and 7.9 cm^2^/y until 15.4 years and 1.3 YPHV, respectively. After these age and maturation breakpoints, the changes in CMA reached a plateau. (*p* = 0.04–0.08). In modeling the CMA trajectories, the model strength (r^2^ = 0.53 and r^2^ = 0.56 for CA and YPHV, respectively) was not as large as the other variables examined.

For TMA, two different patterns were observed. In the CA model (r^2^ = 0.72), two time points of change in the player’s development were estimated at 13.5 and 14.5 years. The growth rate increased after the first breakpoint (increasing from 10.1 cm^2^/y to 43.3 cm^2^/y) but decreased after the second one (changing from 43.3 cm^2^/y to 3.5 cm^2^/y). However, the precision of the estimated slope estimated was weak compared to other BC characteristics: the 95% CI was much larger and overlapped. In the YPHV model (r^2^ = 0.78), the player’s rate of development was estimated at 18.3 cm^2^/y, until 1.2 YPHV, the only breakpoint observed. After this time point, there was no further increase and the trajectory showed a plateau.

### 3.2. BIVA Patterns

When VL and PA were modeled against CA, segmented regression analysis identified two time points of change, estimated at 12.8 and 14.3 years for VL, and at 12.6 and 16.9 years for PA (Figure 2). However, the trajectory patterns were different: in the VL-CA model a decrease in VL values was observed with increasing CA with the greatest loss rate detected between the two breakpoints, and estimated at −76.7 ohm/m/y. However, the PA-CA model suggested a development gain between the two breakpoints, which was estimated at 0.4 °/y (*p* < 0.001), and showed a flat trajectory before 12.6 years (slope *p* = 0.48) and an almost-flat trajectory after 16.98 (slope *p* = 0.05). The model strength was higher in VL (r^2^ = 0.79) than in PA (r^2^ = 0.58).

**Figure 3:**
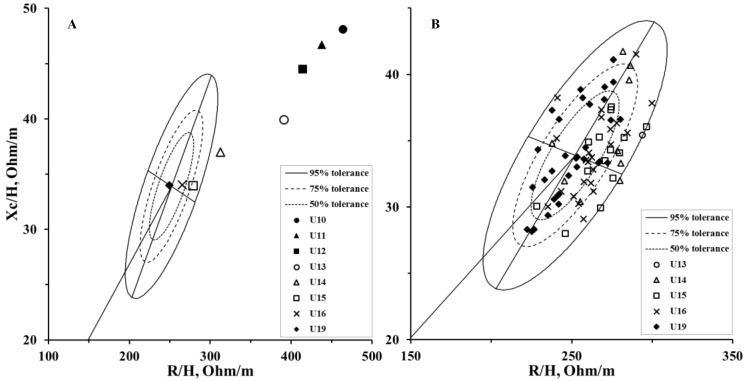
Mean impedance vectors for each age category (**A**) and single vectors (**B**) plotted on the adult soccer players tolerance ellipses [28].

Figure 3 shows the mean vectors of the soccer players grouped by age category (Figure 3A) on the R–Xc graph and the single vectors only for those entered in the reference tolerance ellipses (Figure 3B). In total, 83 players entered the 95° percentile of the tolerance ellipses, including 40.9% (n = 34) from the U19, 28.9% (n = 24) from the U16, 16.8% (n = 14) from the U15, 12.0% (n = 10) from the U14, and 1.2% (n = 1) from the U13 categories.

## 4. Discussion

This study aimed to evaluate the influence of CA and somatic maturation on BIVA patterns and on BC in elite youth soccer players. In order to do so, we identified the breakpoints in a wide range of bioelectric measurements and body composition data. Additionally, vector displacements in the R–Xc graph due to growth were assessed in comparison to elite adult soccer players.

Our results showed non-constant linear regression parameters in the linear predictor and the existence of at least one breakpoint for all examined variables, except for fat-related parameters (FM, %FM, UFA, CFA, and TFA). Most likely, soccer match demands and training adaptations cause an increase in FFM, maintaining low levels of %FM without significant changes. In the general population, %FM decreases with growth, shows a peak around 16 years [33], and then remains constant [34]. Additionally, Agostinete et al. [35] showed that the %FM is higher in young sedentary adolescents than in those who practice sports, without any impact of the maturity status.

The developmental tempo of FFM, TBW and BCM markedly increase at 11.7, 13.4 and 13.6 years, or −2.2, −1.8 and −0.9 YPHV, and disappear (FFM) or subside (TBW, BCM) circa-PHV or post-PHV, e.g., at 15.1, 14.2, and 14.1 years, and at 1.3, 1.3, and 0.44 YPHV, respectively, thus showing asynchronous development. For UMA and TMA, the developmental tempo sped up between 12.8 and 15.1 years, and between 13.4 and 14.5 years, respectively, while constant growth was observed for CMA until 15.4 years. However, no notable accelerated phases were detected when the BC was modeled against YPHV, except for UMA, for which the developmental tempo increased notably between −1.7 and 0.9 YPHV and vanished after the second breakpoint. For all the other BC variables, constant growth rates were observed until slightly after the PHV, when their developmental tempo disappeared. Our results showed that at time points around the PHV period, early somatic maturation was associated with accelerated development in PA and VL/H and BC parameters relative to players who matured late. An increase in the rate of development was observed between 12.8 and 14.3 years for VL/H, and between 12.6 and 16.9 years for PA, with a decreasing trajectory beyond the second breakpoints. More linear progressive trajectories were observed when these two variables were related with somatic maturation. In particular, the developmental tempo of VL increased from −3.9 to 1.1 YPHV and waned after this time point. However, for PA a significant developmental gain started from −1.5 YPHV, and a constant growth rate was observed until advanced post-PHV. Another important finding, given the present sample and analysis approach was related to the better predictive ability of the models (shown by the r^2^ values) with YPHV, rather than CA, providing an explanatory variable for all the BC and bioimpedance parameters except for PA. This is not surprising, given the timing and nature of the adolescent growth spurt (which is typically different among youth), and the asynchronous relationship between different BC and bioimpedance indicators. The enhanced strength of the model might be better explained by the accompanying and direct anthropometric changes that occur close to PHV [25].

The reference tolerance ellipses used to create the R–Xc graph are those proposed by Micheli et al. [28] for elite soccer players. According to the BIVA approach, the 50%, 75% and 95% confidence intervals allow TBW and soft tissues (e.g., BCM and FFM) to be evaluated in relation to reference population-specific ranges. This allows the monitoring and comparison of BC changes [36], looking to achieve the ideal soccer player profile [28]. We observed vector displacements in athletes from the upper right towards the lower left, aimed at the 50th percentile in the R–Xc graph, where the U19 group placed exactly in the ellipse’s center, showing a BC similar to that of the 219 soccer players of the first Italian division measured by Micheli et al. [28]. The mean impedance vectors of the players divided by CA indicate that from the U15 category and on, athletes are positioned inside the tolerance ellipses. However, if we consider the single vectors, even N = 1 U13 and N = 10 U14 athletes are included in the 95% confidence interval. In our opinion, this happens because even when athletes are grouped by CA, as happens in soccer competitions, they may mature earlier than others in the same age category [3]. The BIVA vector represents a simultaneous interpretation of the PA and the VL/H, which reflect the changes in TBW, BCM and FFM. Therefore, as in our case, younger athletes can show advanced biological maturity, and therefore a BC similar to that of adult soccer players. In line with our results, Koury et al. [24] showed that PA is influenced by maturity, while De Paolo [37] showed that vector shortening occurs with growth. This is due to the increase in soft tissue and fluid, which is primarily related to somatic maturation, and thus, also to the CA. Previous studies [14,28] have shown that BIVA patterns also change according to the competitive level, managing to discriminate elite and sub-elite athletes. This is because BC influences performance and therefore athletes who compete in elite categories have less fat (e.g., UFA, TFA, CFA and% FM) and higher BCM and FFM [28,38]. To our knowledge, this is the first study that evaluates the changes in the BIVA patterns and BC in relation to maturity status and CA in elite youth soccer players.

Lastly, if we look at the YPHV, we can see that on average the players were in a stage of advanced or average maturation, which according to Malina et al. [39] precedes the average age of PHV (14.0 years in males) by one year. This occurs in all categories except in the U19 group where the players have reached late maturity (15.5 years). This indicates that in younger groups, selection is more favorable for athletes who have matured early, while this factor may no longer be important as they approach adulthood. Furthermore, the boys selected due to their advanced maturation in the minor categories might then be rejected during the subsequent selection processes.

Future studies should identify non-invasive measurable variables that can be used to categorize maturation status. In this regard, in a cross-sectional study conducted by Backous et al. [40] showed how body height and grip strength can provide important information for classifying physical maturity in boys.

## 5. Conclusions

The assessment of BC may assist in optimizing competitive efficiency and monitoring the success of training regimes for soccer players. We identified transition time points where the influence of age and maturation increased or waned. As our results show, BIVA patterns and BC variables should consider somatic maturation, as young athletes may already present BC characteristics similar to those of adults. In fact, growth and maturation trajectories may occur later in non-selected youth soccer players.

## Figures and Tables

**Table 1:** Descriptive statistics (mean ± SD) of the players grouped by age category.

Variable	U10 N = 26	U11 N = 26	U12 N = 26	U13 N = 41	U14 N = 31	U15 N = 28	U16 N = 29	U19 N = 35
Age (y)	9.9 ± 0.2	10.8 ± 0.3	11.7 ± 0.3	12.8 ± 0.3	13.9 ± 0.2	14.9 ± 0.2	15.5 ± 0.5	17.7 ± 0.5
Weight (kg)	31.6 ± 4.0	31.6 ± 4.6	35.9 ± 5.2	39.7 ± 7.4	48.0 ± 7.5	55.4 ± 5.8	63.8 ± 7.1	67.7 ± 6.0
Height (cm)	131.6 ± 6.2	144.5 ± 5.3	151.1 ± 7.5	152.8 ± 11.1	167.7 ± 8.0	174.0 ± 7.2	176.1 ± 5.9	177.4 ± 5.8
BMI (kg/m^2^)	16.9 ± 1.4	17.1 ± 1.5	18.7 ± 1.7	19.3 ± 2.1	20.7 ± 1.9	21.1 ± 1.0	21.8 ± 1.8	22.0 ± 1.3
Sitting height (cm)	61.1 ± 2.9	71.5 ± 2.9	73.3 ± 3.9	77.9 ± 3.5	83.7 ± 4.1	88.3 ± 3.5	93.2 ± 2.5	88.9 ± 3.6
Leg length (cm)	70.5 ± 5.0	73.0 ± 4.0	77.8 ± 13.8	74.9 ± 10.6	84.0 ± 4.5	85.7 ± 4.7	82.9 ± 4.5	88.5 ± 5.6
YPHV (y)	−3.8 ± 0.3	−3.0 ± 0.3	−2.4 ± 0.5	−1.3 ± 0.5	−0.1 ± 0.6	1.0 ± 0.5	2.0 ± 0.5	2.5 ± 0.5
FM (%)	12.8 ± 2.2	14.0 ± 3.2	15.9 ± 3.9	13.9 ± 4.0	12.3 ± 3.0	10.9 ± 1.9	13.3 ± 2.5	14.7 ± 2.5
FFM (kg)	27.6 ± 3.1	30.8 ± 3.5	33.3 ± 4.0	41.3 ± 5.9	48.5 ± 6.4	58.0 ± 5.8	58.7 ± 5.8	59.0 ± 4.2
UMA (cm^2^)	20.6 ± 3.5	24.6 ± 4.7	25.7 ± 5.0	29.3 ± 5.6	36.3 ± 6.1	45.8 ± 4.7	45.9 ± 7.5	44.3 ± 6.4
UFA (cm^2^)	7.6 ± 1.8	8.6 ± 2.5	9.8 ± 2.8	8.8 ± 3.3	8.5 ± 2.8	7.8 ± 1.9	11.1 ± 2.9	11.5 ± 3.5
CMA (cm^2^)	44.8 ± 5.4	48.0 ± 8.6	54.2 ± 19.1	60.8 ± 9.3	69.1 ± 13.4	65.7 ± 6.9	81.6 ± 12.6	74.6 ± 11.2
CFA (cm^2^)	18.5 ± 5.7	20.5 ± 6.6	24.8 ± 7.1	23.2 ± 7.5	22.9 ± 7.1	19.6 ± 3.4	24.4 ± 5.9	27.1 ± 5.6
TMA (cm^2^)	99.7 ± 12.5	109.8 ± 13.5	119.9 ± 25.7	132.9 ± 21.8	156.6 ± 27.9	183.7 ± 14.2	183.5 ± 22.2	195.4 ± 20.8
TFA (cm^2^)	19.4 ± 4.3	22.3 ± 6.3	23.8 ± 8.8	23.1 ± 7.7	22.6 ± 6.1	21.3 ± 4.2	24.5 ± 7.2	27.7 ± 9.0
R/H (ohm/m)	465.4 ± 53.0	440.8 ± 42.7	416.2 ± 57.4	392.9 ± 55.9	313.5 ± 39.7	276.0 ± 19.4	265.4 ± 17.6	249.7 ± 20.2
Xc/H (ohm/m)	48.3 ± 5.7	46.8 ± 5.6	44.7 ± 6.9	40.1 ± 5.3	37.1 ± 3.9	33.8 ± 3.0	34.1 ± 3.1	34.0 ± 3.8
PA (°)	6.0 ± 0.6	6.1 ± 0.5	6.2 ± 0.5	5.9 ± 0.5	6.8 ± 0.8	7.0 ± 0.5	7.4 ± 0.6	7.8 ± 0.6
VL/H (ohm/m)	467.9 ± 53.1	443.3 ± 42.9	418.6 ± 57.7	395.0 ± 56.1	315.7 ± 39.7	278.1 ± 19.5	267.6 ± 17.6	252.0 ± 20.4
TBW (l)	20.9 ± 2.1	22.9 ± 3.2	25.1 ± 4.1	30.9 ± 3.0	35.5 ± 3.6	42.4 ± 3.6	43.0 ± 2.7	42.8 ± 3.2
BCM (kg)	21.9 ± 1.4	22.4 ± 1.8	23.5 ± 1.8	23.9 ± 2.2	28.7 ± 4.1	31.7 ± 2.0	33.4 ± 2.0	35.7 ± 2.4

Note: BMI = body mass index; YPHV = years from peak high velocity; FM = fat mass; FFM = fat free mass; UMA = upper arm muscle area; UFA = upper arm fat area; CMA = calf muscle area; CFA = calf fat area; TMA = thigh muscle area; TFA = thigh fat area, R/H = resistance standardized for height, Xc/H = reactance standardized for height, PA = phase angle, VL/H = vector length standardized for height, TBW = total body water, BCM = body cell mass.

**Table 2:** Body composition and bioimpedance patterns: development trajectories of the soccer players according to chronological decimal age (years).

	Breakpoints in Development (Years)		Rate of Player Development						
Variable	Breakpoint 1. (95% CI)	Breakpoint 2. (95% CI)	Slope 1 (95% CI)	*p*-Value	Slope 2 (95% CI)	*p*-Value	Slope 3 (95% CI)	*p*-Value	r^2^
FFM	11.7 (11.1; 12.3)	15.1 (14.5; 15.7)	2.1 (−0.4; 4.7)	0.056	7.6 (6.7; 8.5)	<0.001	0.1 (−1.7; 1.8)	0.48	0.84
TBW	13.4 (12.9; 13.9)	14.2 (13.8; 14.6)	2.1 (1.3; 2.8)	<0.001	12.7 (5.0; 20.4)	0.01	1.4 (0.8; 2.0)	<0.001	0.84
BCM	13.6 (13.2; 14.0)	14.1 (13.8; 14.4)	0.7 (0.2; 1.1)	<0.001	14.1 (3.6; 24.6)	<0.01	1.2 (0.8; 1.6)	<0.001	0.83
UMA	12.9 (12.2; 13.5)	15.1 (14.4; 15.8)	2.2 (0.9; 3.6)	<0.001	7.9 (6.0; 9.8)	<0.001	−0.8 (−2.8; 1.2)	0.22	0.72
CMA	15.4 (14.6; 16.2)	-	7.6 (6.5; 8.8)	<0.001	−3.7 (−8.0; 0.5)	0.04	-	-	0.53
TMA	13.5 (12.7; 14.2)	14.5 (13.6; 15.3)	10.1 (5.8; 14.4)	<0.001	43.3 (4.9; 81.6)	0.01	3.5 (0.1; 7.0)	0.02	0.72
VL/H	12.8 (12.1; 13.5)	14.3 (13.9; 14.7)	−19.4 (−29.5; −9.3)	<0.001	−76.7 (−103.5; −49.9)	<0.001	−9.0 (−15.6; −2.5)	<0.01	0.79
PA	12.7 (11.9; 13.4)	16.9 (16.3; 17.5)	−0.1 (−0.2; 0.2)	0.48	0.4 (0.3; 0.5)	<0.001	−0.4 (−0.8; 0.0)	0.055	0.58

Note: CI = confidence interval; FFM = fat free mass; TBW = total body water, BCM = body cell mass; UMA = upper arm muscle area; CMA = calf muscle area; TMA = thigh muscle area; PA = phase angle; VL/H = vector length standardized for height; statistical significance set at *p* < 0.05.

**Table 3:** Body composition and bioimpedance patterns: development trajectories of the soccer players according to years from peak of velocity (years).

	Breakpoints in Development (Years)		Rate of Player Development						
Variable	Breakpoint 1. (95% CI)	Breakpoint 2. (95% CI)	Slope 1 (95% CI)	*p*-Value	Slope 2 (95% CI)	*p*-Value	Slope 3 (95% CI)	*p*-Value	r^2^
FFM	−2.2 (−2.8; −1.6)	1.3 (1.0; 1.6)	3.9 (1.7; 6.0)	<0.001	7.2 (6.4; 8.0)	<0.001	−0.1 (−1.5; 1.5)	0.50	0.91
TBW	−1.8 (−2.4; −1.2)	1.3 (0.6; 2.0)	2.2 (1.0; 3.4)	<0.001	5.1 (4.3; 5.9)	<0.001	1.6 (0.4; 2.8)	<0.01	0.91
BCM	−0.9 (−1.6; −0.2)	0.4 (−0.5;1.3)	1.0 (0.5; 1.5)	<0.001	4.7 (2.9; 6.5)	<0.001	1.9 (1.3; 2.5)	<0.001	0.85
UMA	−1.7 (−2.4; −1.0)	0.9 (0.3; 1.5)	2.2 (0.1; 4.4)	0.02	7.1 (5.4; 8.8)	<0.001	−0.3 (−2.3; 1.7)	0.37	0.76
CMA	1.3 (0.7; 1.9)	-	7.9 (6.6; 9.2)	<0.001	−3.5 (−8.5; 1.5)	0.08	-	-	0.56
TMA	1.3 (0.5; 2.1)	-	18.3 (16.1; 20.5)	<0.001	4.1 (−3.7; 12.0)	0.15	-	-	0.78
VL/H	1.1 (0.6; 1.6)	-	−42.8 (−47.0; −38.6)	<0.001	−9.6 (−23.4; 4.2)	0.09	-	-	0.83
PA	−1.5 (−2.3; −0.7)	-	−0.0 (−0.2; 0.2)	0.44	0.4 (0.3; 0.5)	<0.001	-	-	0.56

Note: CI = confidence interval; FFM = fat free mass; TBW = total body water, BCM = body cell mass; UMA = upper arm muscle area; CMA = calf muscle area; TMA = thigh muscle area; PA = phase angle; VL/H = vector length standardized for height; statistically significance set at *p* < 0.05.
